# The Buffalo Medical College

**Published:** 1882-08

**Authors:** 


### THE BUFFALO MEDICAL COLLEGE.

*Session of 1882–83.*

Among our advertisements will be found the announcement of the Buffalo Medical College for the coming session. Especially, and we may also say painfully, noticeable is the omission of the name of the late Prof. White, which has stood at the head of its faculty for so many years. The strength of his character and the sagacity of his counsel and leadership, guided by an intense interest and ambition for the reputation and success of the college, are a serious loss to this institution, which will more or less influence its future career.

New names, heretofore but little known to its alumni, fill the vacancies and supply the changes thus made. Dr. Mann, a gentleman of wide culture and scholarly attainments, fills the chair of Obstetrics and Gynecology.

The resignation of Prof. Doremus will be a surprise and regret to all who witnessed the energy, zeal and ability he brought to his work. Dr. Witthaus, of New York, has been elected to fill this vacancy and will take up his residence in Buffalo.

The chair of Materia Medica and Hygiene is again filled by the former incumbent, Dr. Stoddard, who has been induced to withdraw his final resignation offered over a year, and to again undertake the self-sacrificing duties of professorial work.

Several new lecturers are appointed, among whom we notice with pleasure our late editorial associate, Dr. Howe, who will give the class the benefit of his ripe experience and acknowledged ability in the department of Ophthalmology.

A most excellent selection is that of Dr. M. B. Folwell, who assumes the lectureship of Dermatology and Syphilis. Dr. Folwell has already established an enviable reputation in this specialty, and is a valuable acquisition to the corps of instructors.

Dr. Richard M. Moore, of Rochester, fills the position of lecturer on Histology.

These changes betoken a liberal policy on the part of the college faculty. The term of lectures has also been lengthened. We look for another step in advance at an early day—the establishment of a graded curriculum of study and a higher standard of acquirements for the degree of Doctor of Medicine.

